# Terpolymerization of Substituted Cycloolefin with Ethylene and Norbornene by Transition Metal Catalyst

**DOI:** 10.3390/polym8030060

**Published:** 2016-02-26

**Authors:** Laura Boggioni, Nella Galotto Galotto, Fabio Bertini, Incoronata Tritto

**Affiliations:** Istituto per lo Studio delle Macromolecole (ISMAC), Consiglio Nazionale delle Ricerche (CNR) Via E. Bassini 15, 20133 Milano, Italy; galotto@ismac.cnr.it (N.G.G.); bertini@ismac.cnr.it (F.B.)

**Keywords:** terpolymerization, norbornene, substituted cycloolefin, metallocenes

## Abstract

Ethylene-norbornene terpolymerization experiments using 5-alkyl-substituted norbornenes (5-pentyl-2-norbornene (C_5_N) and 5-octyl-2-norbornene (C_8_N)) or dicyclopentadiene (DCPD) were conducted with two *ansa*-metallocenes, [Zr{(η^5^-C_9_H_6_)_2_C_2_H_4_}Cl2] (**1**) and [Zr{(η^5^-2,5-Me_2_C_5_H_2_)_2_CHEt}Cl_2_] (**2**), activated by methylaluminoxane (MAO). The terpolymers obtained were investigated in detail by determining the microstructure and termonomer contents by ^13^C NMR, molar masses and thermal properties. Results were compared to those of ethylene (E)-norbornene (N) terpolymerizations with 1-octene. **2**, with lower steric hindrance and a shorter bridge, gave the best activities, termonomer incorporation and molar masses. The size of the substituent in 5-alkyl substituted norbornene also plays a role. C_8_N gives the highest activities and molar masses, while DCPD terpolymers have the highest cycloolefin content. Terpolymers are random; their molar masses, much higher than those in 1-octene terpolymers, are in a range useful for industrial applications. Finally, *T*_g_ values up to 152 °C were obtained. For similar N content, poly(E-*ter*-N-*ter*-C_8_N)s and poly(E-*ter*-N-*ter*-DCPD)s have the lowest and the highest *T*_g_ values, respectively. Thus, the presence of an eight-carbon atom pendant chain in C_8_N increases the flexibility of the polymer chain more than a five-carbon atom pendant chain in C_5_N. The higher rigidity of C_5_N may lead to lower activities and to increasing probability of σ-bond metathesis and chain termination, as evidenced by chain-end group analysis.

## 1. Introduction

Polyolefins, which incorporate cyclic structures into the main chain (COC), are useful materials, since they possess high glass transition temperatures (*T*_g_), low densities, increased moduli and are dimensionally stable. These properties make the materials useful for optical, electrical, packaging and printing applications. They have become available with the developments of *ansa*-metallocenes and well-defined single-site catalysts, which have enabled the design of olefin homo- and co-polymers with well-defined structures [1,2,3,4]. Heterogeneous Ziegler–Natta ethylene (E) polymerization catalyst systems are unable to incorporate cyclic olefins into the polymer chain, while *ansa*-metallocenes and, in particular, zirconocenes can incorporate cyclic olefins into the polyethylene backbone with high activity [5,6,7,8,9,10,11].

The synthesis of poly(ethylene-*co*-norbornene)s with a number of single-site catalysts have attracted great industrial and academic interest [12,13,14,15,16,17,18,19,20,21,22,23,24,25,26,27]. The norbornene (N) content in the main chain can be controlled by varying the catalytic system and by increasing the N concentration in the feed. The resulting material is an amorphous copolymer with high *T*_g_, good transparency and increased heat resistance. Poly(ethylene-*co*-norbornene)s with more than 20 mol % of norbornene are amorphous thermoplastic materials with a broad range of outstanding properties, like excellent moisture barrier, high glass transition temperatures up to 220 °C, thermoformability, stiffness, dead-fold characteristics and chemical inertness. Copolymer properties, which are controlled by cyclic comonomer content, sequence distribution and the stereochemical placement of norbornene units, are tightly connected to the structure of the *ansa*-metallocene employed [14]. One possibility to further widen the application of COC is the introduction of a third monomer. The opportunity of incorporating other olefins (as a third monomer) into the poly(ethylene-*co*-norbornene) backbone may lead to improvements in the mechanical or other physical properties of the polymer. There are only a few reports on terpolymerization of ethylene and norbornene [28,29,30,31]. The introduction of a termonomer, either a linear α-olefin, such as 1-octene (O), or an alicyclic α-olefin, like vinylcyclohexane, has been reported by Marconi *et al.* [29], but a strong decrease of *M*_w_ has been observed for both 1-octene and vinylcyclohexane. It was shown that 1-octene and vinylcyclohexane behave as chain termination/transfer agents, that is they act as reagents that both terminate and facilitate the reinitiation of a growing polymer chain [29,32].

**Figure 1:**
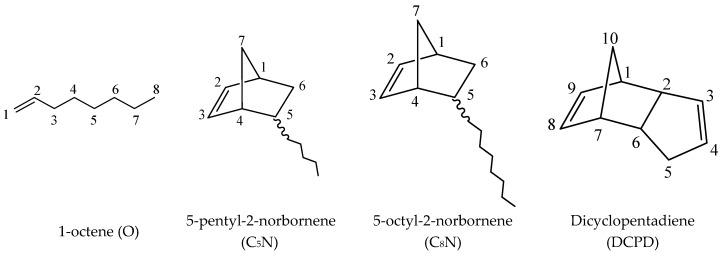
Termonomers selected.

In the present study, we explored the possibility to maintain the advantages of the presence of a long linear alkyl chain branching (that is an increase of chain flexibility with a consequent reduction of brittleness) or of a sterically-demanding cycloolefin within the poly(ethylene-*co*-norbornene) backbone, avoiding chain transfers and termination reactions. We thought to use bridged ring termonomers, such as dicyclopentadiene (DCPD) or alkyl substituted norbornenes (e.g., 5-pentyl-2-norbornene and 5-octyl-2-norbornene) (Figure 1), where the β-hydrogen elimination and transfer reactions, facile with α-olefins, are disfavored. Indeed, a necessary condition for the β-hydride elimination process is the coplanarity of the Ti-C(α)-C(β)-H bond; due to the *endo* position of the β-H atom, the typical four-center transition state cannot be formed. 5-pentyl-2-norbornene and 5-octyl-2-norbornene, which have rather long alkyl substituents, were selected to assess the effect of the alkyl substituents of different sizes on polymer properties, rather than as candidates for commercialization. Dicyclopentadiene is industrially available at a low price [33] and is a representative cyclic diene, which contains both one norbornene unit and one cyclopentene unit; thus, the double bonds not involved in the polymerization reaction can be hydrogenated or be available for post-polymerization reactions and lead to functionalized polymers [34].

**Figure 2:**
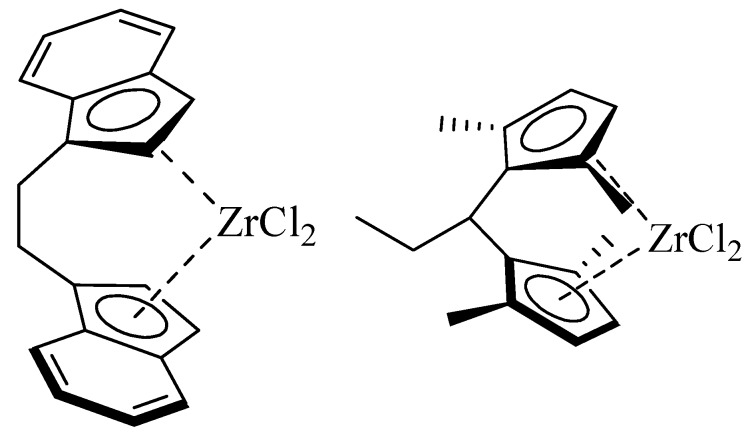
*Ansa-*zirconocene-based precursors used in terpolymerization.

Complexes [Zr{(η^5^C_9_H_6_)_2_C_2_H_4_}Cl_2_] (**1**) and [Zr{(η^5^-2,5-Me_2_C_5_H_2_)_2_CHEt}Cl_2_] (**2**) were selected as catalyst precursors (Figure 2). Complex **1** belongs to the class of *C*_2_-symmetric metallocenes, which is a highly studied group of metallocenes, leading to poly(ethylene-*co*-norbornene) with high molar mass (**1**) [14]. Complex **2** is a *C*_1_-symmetric metallocene, similar to 2,5-dimethylcyclopentadienyl *ansa*-zirconocene complexes, which show excellent activity for the copolymerization of ethylene and bulky comonomers, such as norbornene, due to the absence of any substituent on the α-carbons and the lower steric hindrance of the active site [35]. The aim of this study was to assess the influence of the nature of termonomer and termonomer concentration on polymerization behavior and polymer properties; thus, terpolymerizations were investigated in standard polymerization conditions (*T* = 50 °C, *P* = 1 atm). Polymer analyses by NMR, SEC and DSC were performed to determine terpolymer microstructure, termonomer content, molar masses and thermal properties.

## 2. Materials and Methods

### 2.1. General Considerations and Materials

All manipulations of air-sensitive compounds were performed under nitrogen using standard Schlenk-line and glovebox techniques. Ethylene and nitrogen were purified by passage through BTS-catalysts and molecular sieves. Toluene was fresh-distilled over Na prior to use. Norbornene (purchased from Aldrich, St. Louis, MO, USA) was distilled from Na and used as stock solution in toluene. 1-Octene (98%, Aldrich) was dried over CaH_2_, distilled and stored over molecular sieves. Methylaluminoxane (MAO, 10 wt % solution in toluene, Crompton, Kennesaw, GA, USA) was dried before use (50 °C, 3 h, 0.1 mm Hg) to remove solvent and unreacted trimethylaluminum (TMA). Catalyst complexes were donated by TOPAS Advanced Polymers GmbH, Frankfurt, Germany. C_2_D_2_Cl_4_ was purchased from Cambridge Isotope Laboratories, Inc., Tewksbury, MA, USA and used as received.

### 2.2. Analytical Measurements

NMR spectra were recorded on a Bruker NMR Advanced 400 instrument (400 MHz, ^1^H; 100.58 MHz, ^13^C; pulse angle = 12.50 μs, acquisition time = 0.94 s, delay = 16 s). For NMR analysis, about 100 mg of terpolymer sample were dissolved in C_2_D_2_Cl_4_ in a 10-mm NMR tube and transferred to the spectrometer with the probe head pre-equilibrated at 103 °C. Chemical shifts for ^1^H were referred to internal solvent resonances (5.86 ppm), and chemical shifts for ^13^C were referred to hexamethyldisiloxane (HMDS).

On the basis of the peak areas of carbons in the ^13^C NMR spectra, it was possible to estimate the content of norbornene, ethylene, 5-pentyl-2-norbornene, 5-octyl-2-norbornene and DCPD.

Differential scanning calorimetry (DSC) measurement were carried out on a Perkin-Elmer Pyris 1 instrument. Terpolymers were analyzed in a temperature range from −20 to 200 °C using heating and cooling rates of 20 °C min^−1^. *T_g_* values were recorded during a second thermal cycle. Molar masses (*M*_w_) and the molar mass distribution (*M*_w_*/M*_n_) were determined by high temperature size exclusion chromatography (SEC) by a GPCV 2000 high system from Waters. Measurements were carried out in *o*-dichlorobenzene at 145 °C; a polystyrene standard calibration was used.

### 2.3. 5-Alkyl-2-Norbornene Synthesis

Norbornene, as well as alkyl-norbornene were used after distillation. 5-pentyl-2-norbornene (C_5_N) and 5-octyl-2-norbornene (C_8_N) were synthesized by the Diels–Alder reactions of cyclopentadiene, formed *in situ* via the thermal retro Diels–Alder reaction of dicyclopentadiene, with the corresponding 1-alkenes (1-heptene and 1-decene) [36]: mole ratio, CPD:1-heptene = 1:2, at 170 °C, for 20 h, at a pressure of 1 bar of N_2_; mole ratio, CPD:1-decene = 1:2, at 200 °C, for 24 h, at a pressure of 2 bar of N_2_; 5-pentyl-2-norbornene, *T*_b_ = 68 °C/3 mm Hg; and 5-octyl-2-norbornene, *T*_b_ = 140 °C/ 4 mm Hg.

### 2.4. Polymerization Procedure

Terpolymerization reactions were carried out in a 250-mL glass reactor. Before starting the polymerization reaction, the glass reactor was evacuated three times for 15 min at room temperature and filled with nitrogen. The glass reactor was filled with toluene, norbornene, MAO and the appropriate amount of a third monomer (1-octene, 5-pentyl-2-norbornene, 5-octyl-2-norbornene and DCPD). After thermal equilibration (50 °C), the system was saturated with ethylene at atmospheric pressure. The reaction was initiated by the injection of the catalyst dissolved in toluene. The terpolymerization was quenched by the addition of ethanol acid, after 15/20 min, and precipitated in ethanol. The terpolymer products were left stirring overnight and then collected by filtration and dried under vacuum at 70 °C.

### 2.5. Monomers Content Determination

The monomer content in the co- and 1-octene terpolymers was calculated on the basis of [29]. The monomer content in all of the other terpolymers was calculated on the basis of the following equations and of the assignments reported in Table 1.

In particular, Equations (1) to (3) give the termonomer content in mol %:

(1) $$N\left(\text{mol }\%\right)=\frac{I_{N}}{I_{N}+I_{E}+I_{C_{X}N}}$$

where X = 5 or 8.

(2) $$C_{X}N\left(\text{mol }\%\right)=\frac{I_{C_{X}N}}{I_{N}+I_{E}+I_{C_{X}N}}$$

where X = 5 or 8.

(3) $$E\left(\text{mol }\%\right)=\frac{I_{E}}{I_{N}+I_{E}+I_{C_{X}N}}$$

where X = 5 or 8.

It is possible to divide the spectra of poly(E-*ter*-N-*ter*- C_5_N) and poly(E-*ter*-N-*ter*-C_8_N) into three regions (A, B and C).

Region A, from 12.00 and 21.00 ppm, is where the methyl (CH_3_) and the methylene (CH_2_) *exo/endo* carbon atoms of the pentyl or octyl unit (1 B_5_ and 2 B_5_ for C_5_N or 1 B_8_ and 2 B_8_ for C_8_N) appear at 12.03 and 12.05 ppm and 20.67 and 20.90 ppm, respectively. Since this window of the spectra is free from other signals, it allows us to calculate the molar concentration of alkyl substituted norbornene in terpolymers according to Equation (4).

(4) $$A=\;I_{C_{X}N_{\;}}=\;\frac{1}{2}\sum^{\text{​}}\left(I_{1B_{X}}+I_{2B_{X}}\right)$$

where X = 5 or 8

Region B, from 25.00 to 36.25 ppm, is where the signals diagnostic for ethylene units, the signals of norbornene (C5, C6 and C7), the signals of 5-pentyl-2-norbornene (C6’, C7’ and 3 B_5_, 4 B_5_, 5 B_5_) or of 5-octyl-2-norbornene (C6’,C7’, 3 B_8_, 4 B_8_, 5 B_8_, 6 B_8_, 7 B_8_ and 8 B_8_) appear. Thus, we can write Equation (5):

(5) $$B=3I_{N}+XI_{C_{X}N}+2I_{E}$$

where X = 5 or 8

Region C, from 36.26 to 50.00 ppm, where the signals of C1/C4, C2/C3 carbon atoms of norbornene [29] and the C1’/C4’, C2’/C3’ of the alkyl substituted norbornenes, as well as C5’ resonate, allows us to write Equation (6).

(6) $$C=4I_{N}+5I_{C_{X}N}$$

where X = 5 or 8

Then, the N and E molar concentration can be calculated according to Equations (7) and (8), respectively.

(7) $$I_{N\;}=\;\frac{1}{4}\left(C-5A\right)$$

(8) $$I_{E\;}=\;\frac{1}{2}\left(B-3I_{N}-XA\right)$$

where X = 5 or 8.

The content of DCPD was calculated on the basis of Equation (9). The molar concentration of DCPD was quantitatively evaluated from Equation (10), on the basis of the integration values of DCPD diagnostic signals, such as C3’ at 130.64 ppm, C4’ at 128.88 ppm, C2’ at 51.72 ppm, C6’ at 40.91 ppm, C10’ at 34.35 ppm and C5’ at 33.4 ppm [29].

(9) $$DCPD\;\left(\text{mol }\%\right)=\;\frac{I_{DCPD}}{I_{DCPD}+I_{E}+I_{N}}$$

(10) $$I_{DCPD}=\frac{1}{6}\sum^{\text{​}}I_{C3'}+I_{C4'}+I_{C2'}+I_{C6'}+I_{C10'}+I_{C5'}$$

It is possible to calculate N and E molar concentration from the A and B regions of the spectra of poly(E-*ter*-N-*ter*-DCPD).

In Region A, from 25.00 to 30.20 ppm, the C5, C6 signals of the N and CH_2_ signals of ethylene units are visible.

(11) $$A=2I_{N}+2I_{E}$$

In Region B, from 42.10 to 50.00 ppm, the C2, C3 signals of N and those of C8’ and C9’ of DCPD appear.

(12) $$B=2I_{N\;}+\;2I_{DCPD}$$

Thus, from Equations (13) and (14), the N and E molar concentration can be calculated.

(13) $$I_{N\;}=\;\frac{1}{2}\left(B-2I_{DCPD}\right)$$

(14) $$I_{E=}\frac{1}{2}\left(A-B+2I_{DCPD}\right)$$

## 3. Results and Discussion

Terpolymerization reactions were investigated by using two representative *ansa*-zirconocenes, **1** and **2**, activated with methylaluminoxane (MAO). Terpolymerizations were carried out at a temperature of 50 °C. The [N]/[E] ratio ranges between 2, 4 and 8, while the termonomer concentration was kept constant and equal to that of ethylene. The 5-pentyl-2-norbornene and 5-octyl-2-norbornene termonomers have been synthesized from the Diels–Alder reaction as a mixture of *endo* and *exo* isomers [36,37].

Microstructure and norbornene content in the copolymers were determined by means of ^13^C NMR spectroscopy. Molar masses and glass transition temperatures were determined by SEC and DSC measurements, respectively. Selected results of the terpolymerization reactions of E and N with 5-pentyl-2-norbornene, 5-octyl-2-norbornene and DCPD by means of MAO-activated metallocenes **1** and **2** are reported in Table 2. For the comparison, the results of ethylene-*co*-norbornene copolymerization, as well as those of ethylene-*ter*-norbornene-*ter*-1-octene under identical conditions are also included in the table.

### 3.1. Activities

The activities in ethylene-*co*-norbornene polymerization are very similar for the two catalytic systems (Entries 1, 2, 8–10 in Table 2). As expected, the activities slightly drop for both catalytic systems by increasing the norbornene amount in the feed. Moreover, Catalyst **2** shows good activity also for a high amount of N in the feed (Entry 10 in Table 2).

**Figure 3:**
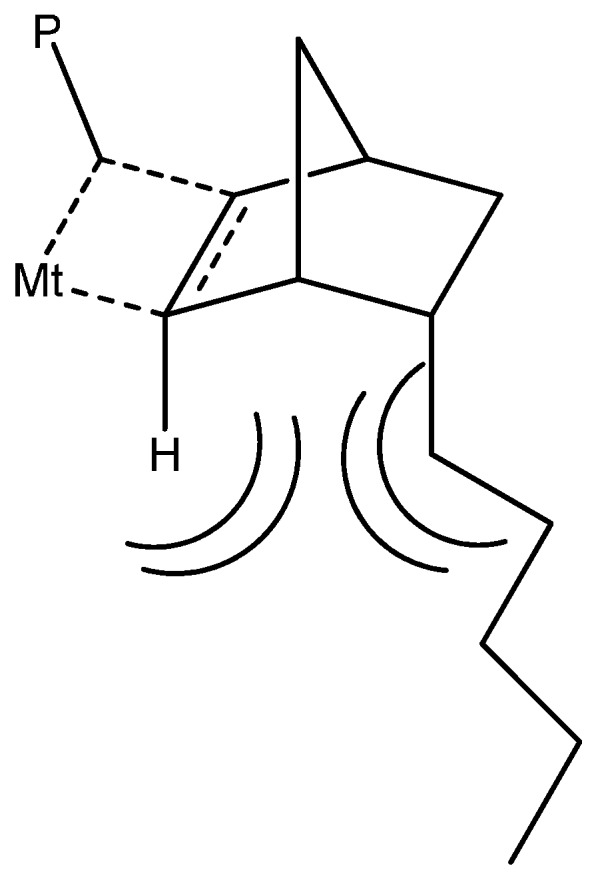
Steric compression in the insertion of *endo* 5-alkylsubstituted norbornene.

In terpolymerization, with Catalyst **1**, the activities for C_5_N, C_8_N and DCPD are very similar at an [N]/[E]/[termonomer] molar feed ratio of 4/1/1 (Table 2, Entries 5–7) and are low, especially when compared to those of 1-octene (Table 2, Entry 3). Catalyst **2** is much more active than Catalyst **1** with all three termonomers. In particular, in the poly(E-*ter*-N-*ter*-C_5_N) synthesis for the feed ratio of 4/1/1, the addition of C_5_N in the polymerization reaction gives the lowest polymerization activity with both Catalysts **1** (Entry 5 in Table 2) and **2** (Entry 13 in Table 2). Regarding the synthesis of poly(E-*ter*-N-*ter*-C_8_N), the addition of alkyl norbornene reduces to a certain degree the activity of Catalyst **2** (Entries 14 and 17 *vs*. 9 and 10 in Table 2), this reduction is much stronger with Catalyst **1** (Entries 6 and 2 in Table 2). This behavior may be partially related to the *exo*, *endo* isomer composition of C_5_N, C_8_N monomers. In general, the *endo* 5-alkyl norbornene isomers are known to be less reactive than the *exo* isomers [38,39,40] because of steric compression, as depicted in Figure 3, where the preferred *cis-exo* addition of norbornene to the Mt-P bond is shown.

In the terpolymerization experiments with DCPD activities intermediate to those with the other termonomers are observed, Catalyst **2** is always more active than **1** under identical conditions (activities of Entries 15 *vs*. 7 in Table 1).

### 3.2. Microstructure

**Figure 4:**
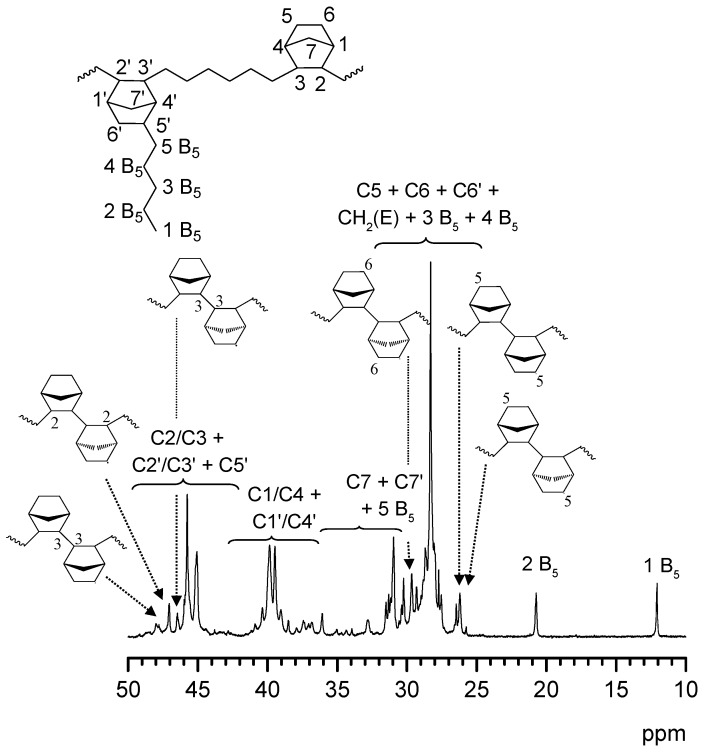
^13^C NMR spectrum (108.58 MHz, C_2_D_2_Cl_4_, 103 °C) of poly(E-*ter*-N-*ter*-C_5_N); sample prepared by **2** (Table 2, Entry 13).

**Figure 5:**
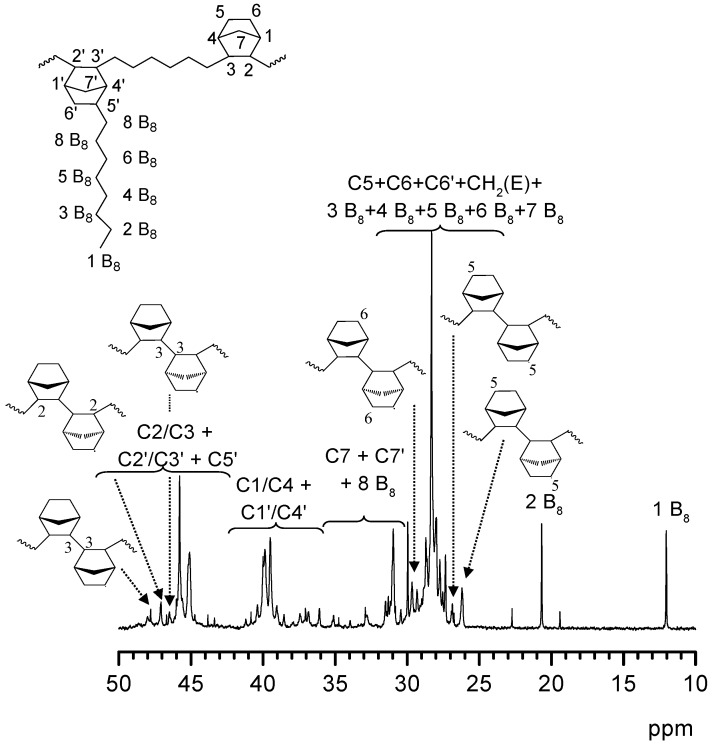
^13^C NMR spectrum (108.58 MHz, C_2_D_2_Cl_4_, 103 °C) of poly(E-*ter*-N-*ter*- C_8_N); sample prepared by **2** (Table 2, Entry 14).

**Figure 6:**
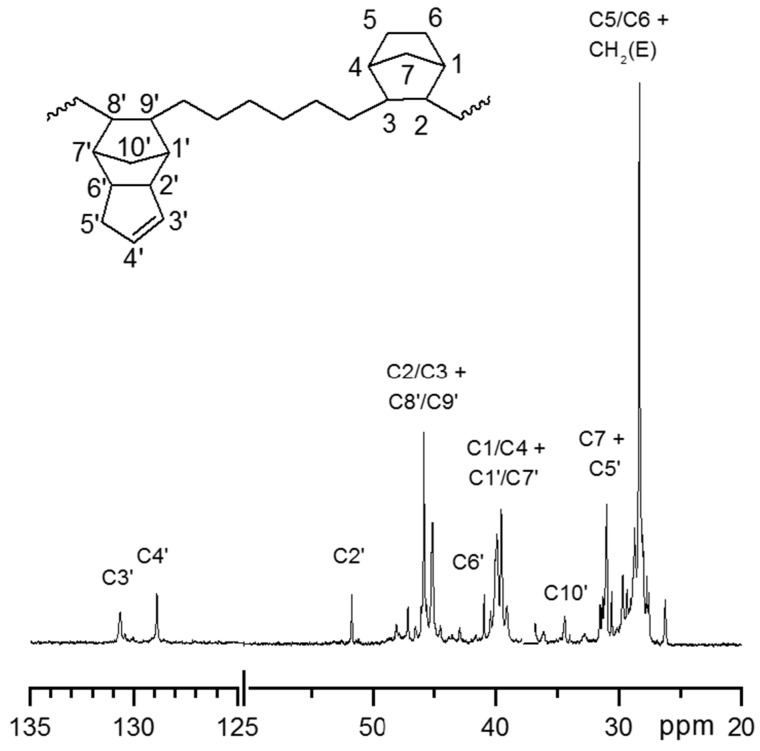
^13^C NMR spectrum (108.58 MHz, C_2_D_2_Cl_4_, 103 °C) of poly(E-*ter*-N-*ter*-DCPD); sample prepared by **2** (Table 2, Entry 15).

Terpolymers were analyzed by ^13^C NMR spectroscopy to evaluate monomer content and eventually terpolymer microstructure. The ^13^C NMR spectra of poly(E-*ter*-N-*ter*-C_5_N), poly(E-*ter*-N-*ter*-C_8_N) and poly(E-*ter*-N-*ter*-DCPD) performed using catalyst **2** are depicted in Figure 4, Figure 5 and Figure 6, respectively. The spectra of samples prepared from **1** are reported in the supporting information. A general structure, for terpolymers, along with the labeling of carbon atom, based on common nomenclature used in literature, is also included in the figures.

The peak assignments, in Table 1, were achieved taking into account DEPT experiments and literature data on poly(E-*co*-N) [20,21,22,23,24,25].

The content of ethylene, norbornene, 5-pentyl-2-norbornene (C_5_N) or 5-octyl-2-norbornene (C_8_N) in the poly(E-*ter*-N-*ter*-C_5_N) and poly(E-*ter*-N-*ter*-C_8_N) was calculated on the basis of Equations (1) to (8) (see the methods and materials part).

The methyl (CH_3_) and the methylene (CH_2_) *exo/endo* carbon atoms of the pentyl or octyl unit (1B_5_ and 2B_5_ for C_5_N or 1B_8_ and 2B_8_ for C_8_N), which appear at 12.03 and 12.05 ppm and 20.67 and 20.90 ppm, respectively, in a window of the spectra free from other signals, allowed calculating the molar concentration of alkyl-substituted norbornene in terpolymers according to Equation (4).

The C5’ chemical shift carbon atom could resonate between 36.10 and 50.00 ppm and overlap the signals of C1/C4 and C2/C3 carbon atoms of norbornene [20,21,22,23,24,25], as well as the C1’/C4’, C2’/C3’ of the alkyl-substituted norbornenes. The comparison between the sum of the integrals of the peaks of the signals between 36.10 and 42.00 ppm and between 42.10 and 50.00 ppm allows us to say that the C5’ might resonate in the area between 42.10 and 50.00 ppm.

The N molar concentration can be calculated according to Equation (7). The E molar concentration can be calculated according to Equation (8), which accounts for the overlapping of signals diagnostic for ethylene units, in the range from 25.00 to 36.25 ppm, with 5-pentyl-2-norbornene (C6’, C7’ and 3 B_5_, 4 B_5_, 5 B_5_) or 5-octyl-2-norbornene (C6’, C7’, 3 B_8_, 4 B_8_, 5 B_8_, 6 B_8_, 7 B_8_ and 8 B_8_) and norbornene signals (C5, C6 and C7) (see the methods and materials part).

The molar concentration of DCPD was quantitatively evaluated from Equation (10), on the basis of integration values of diagnostic signals, such as C3’ at 130.64 ppm, C4’ at 128.88 ppm, C2’ at 51.72 ppm, C6’ at 40.91 ppm, C10’ at 34.35 ppm and C5’ at 33.4 ppm [41]. The C3’ and C4’ signals give evidence of DCPD insertion: the presence of unreacted cyclopentene units indicates that copolymerization proceeds only through insertion of the norbornene moiety. Finally, no crosslinking is observed, as shown by the similar peak areas of the signals diagnostic of DCPD (for example, the C3’ and C4’ peak areas are similar to those of C2’ and C10’,) as well as by the solubility of the polymer samples.

It is worth pointing out that *co*- and *ter*-polymers containing around 50 mol % of cyclic units are random and not alternating, as shown by the signals diagnostic of *meso*-*meso* NN sequences (C5 at 26.24 ppm, C6 at 29.68 ppm, C3 46.50 ppm, C2 at 47.08), by *rac*-*rac* NN sequences (C5 at 27.58 ppm, C3 48.07 ppm) and by the presence of isolated EENEE sequences (e.g., C7 at 30.89 ppm, C1 and C4 at 39.54 ppm).

As in the copolymerization, the N content in the terpolymers increases with increasing N/E molar ratio in the feed. The presence of a termonomer affects the incorporation of N in the polymer chain, especially for Catalyst **1**. Indeed, norbornene content in the terpolymers (Entries 5–7) is clearly much lower than that of Entry 2. This effect, due to termonomer competition with norbornene, is less evident in terpolymers prepared with Catalyst **2** (see N content of Entries 11, 13–15 *vs*. Entry 9).

Regarding termonomer incorporation, the 5-pentyl-norbornene (C_5_N) content in the terpolymers was found to be between 3.4 and 6.7 mol % (Entries 5, 13 and 16 in Table 2). Catalyst **2** incorporates more termonomer than Catalyst **1** for the same N/E/C_5_N feed ratio (Entries 13 and 5 in Table 2). With Catalyst **2**, the highest termonomer incorporation can be obtained in the presence of C_8_N at an [N]/[E] molar feed ratio of 4/1 (Entry 14). It is worth pointing out that C_8_N incorporation is preferred over that of C_5_N; this is only in part a consequence of the *exo*/*endo* ratio of the two monomers [42]. As far as DCPD is concerned, the termonomer incorporation ranges between 3.6 and 6.6 mol % (Table 2). By increasing the molar ratio of norbornene and ethylene in the feed in the composition range of [N]/[E] of four and eight, the incorporation level of all termonomers decreases, as expected. Thus, the highest incorporation of norbornene in the polymer chain is observed in the presence of DCPD and the lowest in the presence of C_8_N with Catalyst **2**. The total cycloolefin content (N plus termonomer) in terpolymers prepared with Catalyst **2** is slightly above the N content of the reference entry, except for the C_8_N terpolymerization. Indeed, of the three termonomers, C_8_N is the one that competes more effectively with norbornene, while terpolymers with DCPD have the highest total cycloolefin content. No crosslinking is observed for the DCPD concentration used, this is probably related to the lower reactivity of cyclopentene moiety with respect to the norbornene moiety of DCPD and to the steric hindrance of the active center, bearing the growing (E-*co*-N) polymer chain as the ligand.

### 3.3. Molar Masses

A comparison of molar masses, determined by SEC, of terpolymers obtained by both **1** and **2** showed that poly(E-*ter*-N-*ter*-C_8_N) and poly(E-*ter*-N-*ter*-DCPD) have high molar masses, though somewhat lower than those of poly(ethylene-*co*-norbornene) obtained under similar conditions, but much higher than those determined for the poly(ethylene-*ter*-norbornene-*ter*-1-octene). Molar masses with Catalyst **2**, as catalytic activities, are higher than those obtained with Catalyst **1**. Molar masses of terpolymers in the presence of C_5_N obtained with Catalyst **2** are significantly lower than those of the other terpolymers (Entries 13 and 16 in Table 1) and closer to the molar masses of poly(ethylene-*ter*-norbornene-*ter*-1-octene). The apparent high *M*_w_ value of Entry 5, with Catalyst **1**, is due to the broad molar mass distribution, probably caused by the very low yield. These observations are more evident by comparing the polymerization degrees, calculated from the following equation:

1/DP = *M*_0_/*M*_n_ (15)

where *M*_0_ and *M*_n_ are the molar mass of the monomer and the number average molar mass, respectively. For a terpolymer, as a first approximation, we take the weighted average of the monomer mass by using the following relationship:

*M*_0_ = *x_E_*·*M_E_ + x_N_*·*M_N_ + x_ter_*·*M_ter_* (16)

where *x_E_*, *x_N_* and *x_ter_* are the molar fractions and *M_E_*, *M_N_* and *M_ter_* are the molar masses of the ethylene, norbornene and termonomer used, respectively.

### 3.4. Chain End Analysis

**Figure 7:**
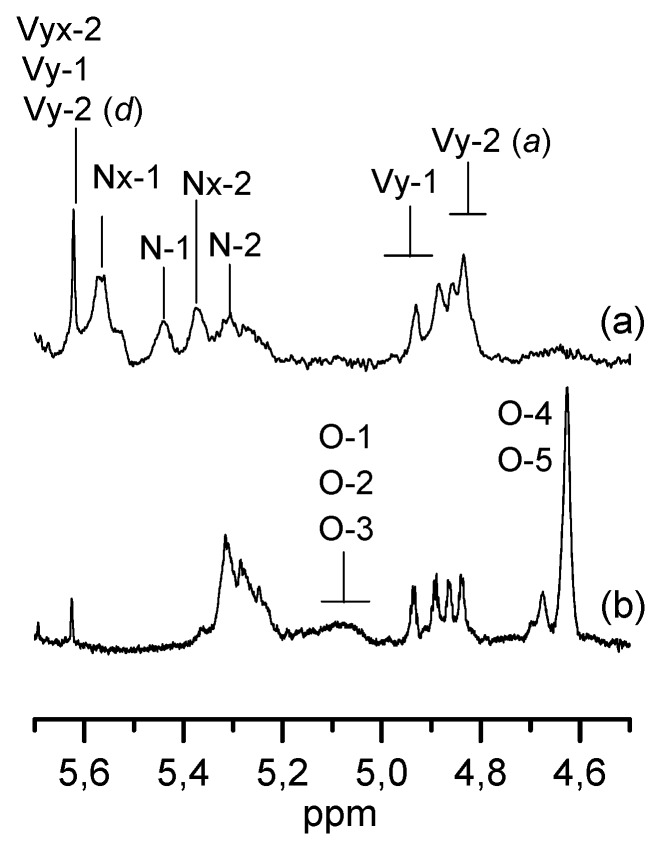
Expansions of the region between 4.4 and 5.7 ppm of the ^1^HNMR spectra (400 MHz, C_2_D_2_Cl_4_, 103 °C) of polymers prepared by **2** at the feed ratio of N/E/termonomer of 4/1/1: (**a**) poly(E-*ter*-N-*ter*-C_8_N); and (**b**) poly(E-*ter*-N-*ter*-octene).

**Figure 8:**
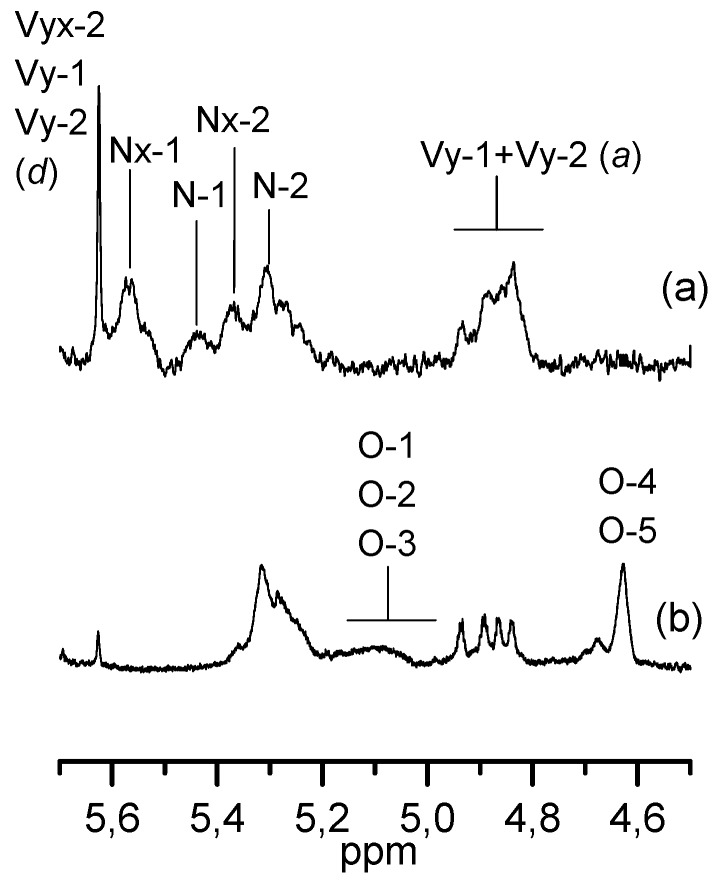
Expansions of the region between 4.4 and 5.7 ppm of the ^1^HNMR spectra (400 MHz, C_2_D_2_Cl_4_, 103 °C) of polymers prepared by **2** at the feed ratio of N/E/termonomer of 8/1/1: (**a**) poly(E-*ter*-N-*ter*-C_8_N); and (**b**) poly(E-*ter*-N-*ter*-octene).

**Figure 9:**
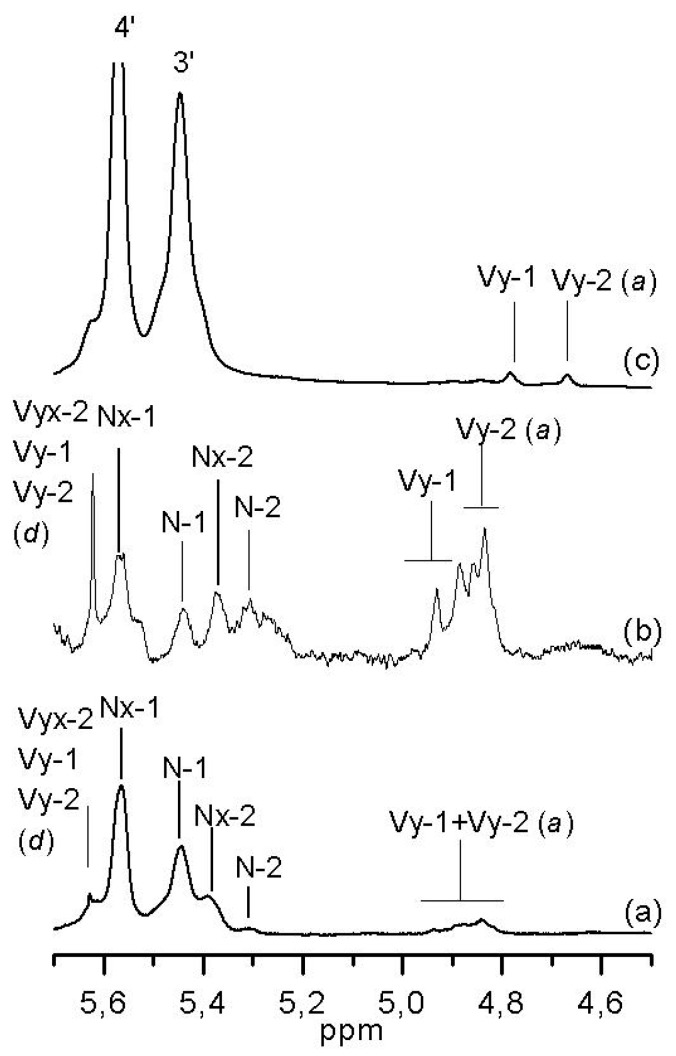
Expansions of the region between 4.4 and 5.7 ppm of the ^1^HNMR spectra (400 MHz, C_2_D_2_Cl_4_, 103 °C) of polymers prepared by **2** at the feed ratio of N/E/termonomer of 4/1/1: (**a**) poly(E-*ter*-N-*ter*-C_5_N); (**b**) poly(E-*ter*-N-*ter*-C_8_N); and (**c**) poly(E-*ter*-N-*ter*-DCPD).

**Figure 10:**
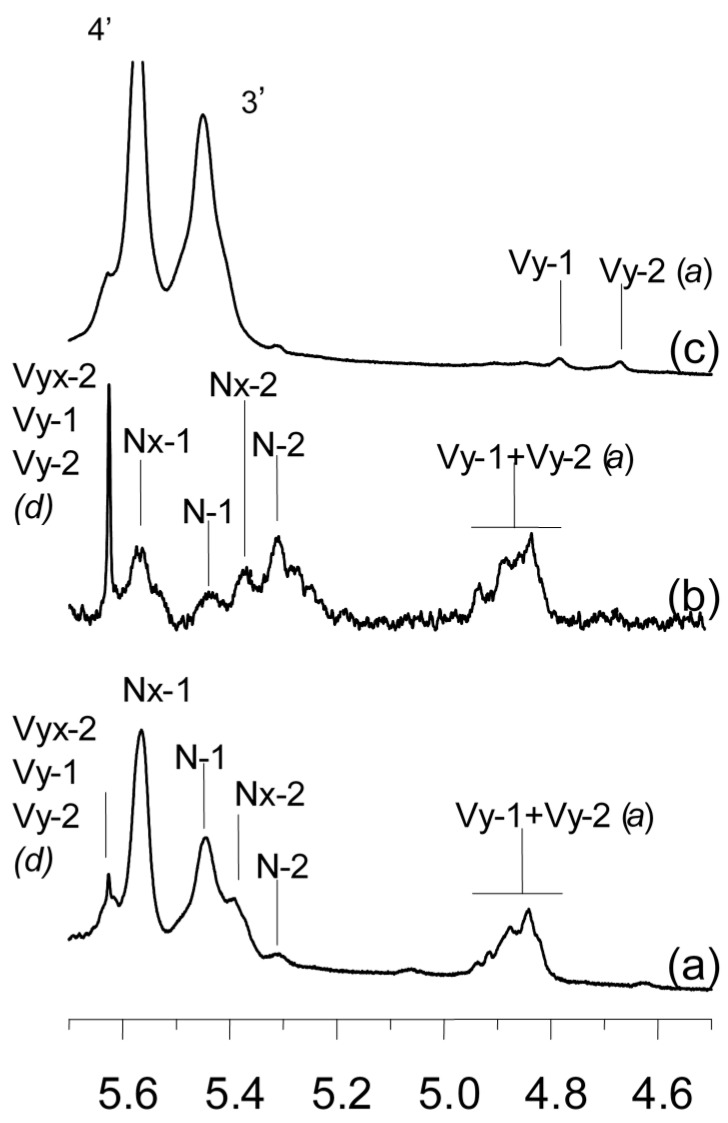
Expansions of the region between 4.4 and 5.7 ppm of the ^1^HNMR spectra (400 MHz, C_2_D_2_Cl_4_, 103 °C) of polymers prepared by **2** at the feed ratio of N/E/termonomer of 8/1/1: (**a**) poly(E-*ter*-N-*ter*-C_5_N); (**b**) poly(E-*ter*-N-*ter*-C_8_N); and (**c**) poly(E-*ter*-N-*ter*-DCPD).

The chain end analysis of the terpolymers can provide direct evidence for the reactions responsible for chain growth termination in the presence of different monomers and catalytic systems. In a given catalytic system, the possible chain transfer pathways are primarily indicated by the relative amount of typical unsaturated end group signals, originating from the first event of β-H transfer to the metal or to the monomer. Figure 7 and Figure 8 show proton spectra expansions of the olefinic region of poly(E-*ter*-N-*ter*-1-octene) and poly(E-*ter*-N-*ter*-C_8_N) obtained with **2** at the N/E/termonomer ratios of 4/1/1 and 8/1/1, respectively, while Figure 9 and Figure 10 show proton spectra expansions of the olefinic region of poly(E-*ter*-N-*ter*-C_5_N), poly(E-*ter*-N-*ter*-C_8_N) and poly(E-*ter*-N-*ter*-DCPD) obtained with **2** at the N/E/termonomer ratios of 4/1/1 and 8/1/1, respectively.

**Scheme 1:**
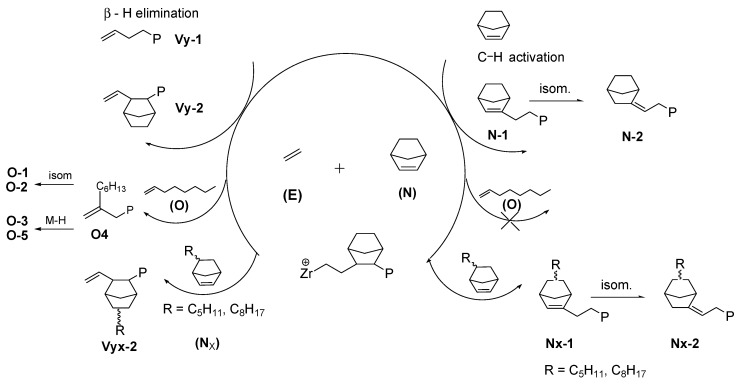
Possible paths leading to terpolymer chain unsaturations in Table 3.

The spectra show the existence of various structural types of olefinic double bonds, associated with the presence of both terminal and internal unsaturations. The resonances of all of these structural units, reported in Table 3, were attributed on the basis of a comparison with those reported in the literature [29,30,31,32,33,34,35,36,37,38,39,40,41,42,43]. Scheme 1 describes the possible paths leading to terpolymer chain unsaturation with a last-inserted E, N, O or Nx (C_5_N or C_8_N) unit.

The comparison of expansions of the olefinic region of the proton spectra of poly(E-*ter*-N-*ter*-1-octene) and poly(E-*ter*-N-*ter*-C_8_N) in Figure 7 and Figure 8 is interesting since it clearly shows the presence of signals designated as O-1, O-2, O-3, O-4 and O-5 chain end groups originating from terminations after the last inserted 1-octene units in the spectra of poly(E-*ter*-N-*ter*-1-octene). They are similar to those of E-O copolymers reported in the literature [44]. The signals at 4.73 and 4.68 ppm attributed to vinylidene structures O-4 and O-5, related to the β-H elimination after 1-octene insertion, are quite strong, while the broad multiplet centered at 5.09 ppm, which includes internal unsaturation O‑1, O-2 and O-3, is rather small.

The signals in the range between 4.8 and 4.93 ppm are characteristic of the vinyl chain end structure Vy-1 and Vy-2, formed by chain termination via β–H elimination after ethylene insertion. The multiplets centered at 5.3 ppm include unsaturated norbornenyl chain start N-1 and N-2, originated by C-H bond activation of a norbornene unit and further isomerization [43].

In the spectra of poly(E-*ter*-N-*ter*-C_8_N), in addition to the signals of vinyl chain end structures Vy-1, Vy-2, and of norbornenyl N-1 and N-2 groups, new signals related to vinyl chain end Vyx-2 and norbornenyl Nx-1 and Nx-2 appear due to the presence of a substituted norbornene as the penultimately or ultimately inserted unit before termination.

In Figure 9 and Figure 10, the spectra of poly(E-*ter*-N-*ter*-C_5_N) (Figure 9a) show strong signals in the range 5.30 to 5.57 ppm ascribed to the norbornene and 5-pentyl-2-norbornene structures Nx-1, Nx-2, N-1 and N-2. In the spectra of poly(E-*ter*-N-*ter*-C_8_N) (Figure 9b and Figure 10b), the amount of vinyl chain end groups (Vy-1,Vy-2 and Vyx-2) is similar to that of the norbornene groups (Nx**-**1, Nx-2, N-1 and N**-**2). Finally, the ^1^H NMR analysis of poly(E-*ter*-N-*ter*-DCPD) evidences only the presence of a small amount of Vy-1 and Vy-2; the great olefinic signals arise from the double bonds of the inserted unit. Worth noting is the great amount of Nx-1 and Nx-2 chain end groups in poly(E-*ter*-N-*ter*-C_5_N). This indicates that termination via C-H bond activation, *i.e.*, σ–bond metathesis, becomes more accessible when the polymerization reaction is slow (see Scheme 1). Fink *et al* [45] demonstrated that σ–bond metathesis is responsible for the C7 linkage in polymerization of norbornene with only *meso* linkages, so that σ–bond metathesis becomes a competitive reaction pathway, especially in a sterically-demanding environment.

These results reflect the relative importance of the various chain transfer pathways that can occur with the different termonomers and explain the different molar masses of the terpolymers obtained.

### 3.5. Thermal Analysis

**Figure 11:**
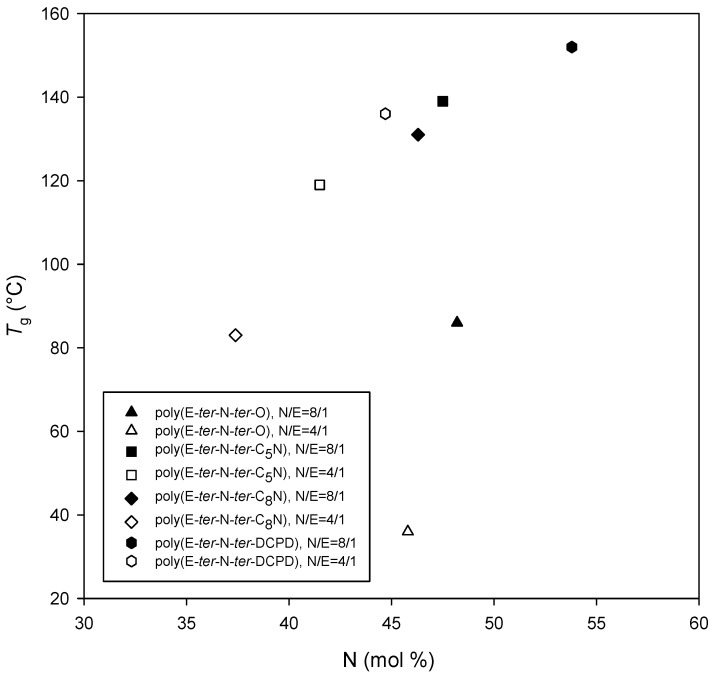
Plot of the glass transition temperature (*T*_g_) *versus* norbornene content in poly(E-*ter*-N-*ter*-Octene), poly(E-*ter*-N-*ter*-C_5_N), poly(E-*ter*-N-*ter*-C_8_N) and poly(E-*ter*-N-*ter*-DCPD) obtained with **2**.

DSC thermal analysis of terpolymers prepared with both Catalysts **1** and **2** showed a single, well-defined glass transition event, while no endothermic peak, corresponding to the melting of any crystalline phase, was observed. This observation implies a homogeneous terpolymer microstructure with completely amorphous morphology. It is evident that glass transition temperatures were clearly a function of N content in the terpolymer and rapidly increase with increasing content of cyclic units, as expected from the previous results of poly(E-*co-*N). Glass transition temperatures ranged from 64 °C for terpolymers with low N content to 152 °C for terpolymers with the highest N content. *T*_g_ values of terpolymers from **2** in the various series were higher compared to those prepared with **1**, owing to the greater N incorporation capability of Catalyst **2**. In Figure 11, *T*_g_ values of the terpolymers prepared by Catalyst **2** were plotted as a function of N incorporation level estimated by ^13^C NMR spectra. It is clear that termonomers also affect *T*_g_ values. Those containing the more rigid DCPD have the highest *T*_g_ values. Among the 5-alkyl-substituted norbornene terpolymers, those containing 5-octyl-2-norbornene show the lowest *T*_g_s; this could be due to the presence of the eight carbon atom pendant chain and indicates an increase in flexibility of the polymer chain. When the eight carbon atom pendant chain is directly linked to the main chain as in poly(ethylene-*ter*-norbornene-*ter*-1-octene), the copolymer chain is even more flexible, and the *T*_g_s are much lower (e.g., Entry 12). It is worth recalling that 1-octene produces also a strong reduction of molar masses.

## 4. Conclusions

A deep investigation of E-N terpolymerizations with the alkyl-substituted norbornene 5-pentyl-2-norbornene (C_5_N) and 5-octyl-2-norbornene (C_8_N) or dicyclopentadiene (DCPD) was conducted with two different *ansa*-metallocene compounds, [Zr{(η^5^-C_9_H_6_)_2_C_2_H_4_}Cl_2_] (**1**) and [Zr{(η^5^-2,5-Me_2_C_5_H_2_)_2_CHEt}Cl_2_] (**2**), in the presence of MAO. The results were compared to those of E-N terpolymerizations with 1-octene under the same conditions. The 2,5-dimethylcyclopentadienyl *ansa*-zirconocene **2** gave the best results in terms of activities, termonomer incorporation and molar masses in the terpolymerization of ethylene, norbornene and hindered substituted norbornene termonomers. Indeed, good activities in the terpolymerization with Catalyst **2**, especially for 5-octyl-2-norbornene, were achieved. The better catalytic performance of **2** compared to **1** in these terpolymerizations, where monomers and the growing polymer chain are quite bulky, could be explained by the lower steric hindrance of the active site of 2, due to the absence of any substituent on the α-carbons, and its shorter –CH(Et)– bridge. All terpolymers are random, as evidenced by ^13^C NMR analysis and DSC experiments. Among the three termonomers, C_8_N is the one that gives also the highest molar masses and competes more effectively with norbornene, while terpolymers with DCPD have the highest N and total cycloolefin content. As far as molar masses are concerned, terpolymer molar masses, much higher than those in 1-octene terpolymers, are in a range useful for industrial applications, though somewhat lower than those of E-N copolymers.

The ^1^H NMR analysis revealed a higher amount of Nx-1 and Nx-2 chain end groups, obtained via σ-bond metathesis, which account for the activities and molar masses observed for poly(E-*ter*-N-*ter*-C_5_N).

Finally, *T*_g_ values ranging from 64 to 152 °C have been obtained. For a similar N content, terpolymers containing 5-octyl-2-norbornene have the lowest *T*_g_; those containing DCPD present the highest *T*_g_ values. The size of the substituent in 5-alkyl-substituted norbornene plays a role. The presence of the eight carbon atom pendant chain increases the flexibility of the polymer chain more than the five carbon atom pendant chain, as evidenced by the *T*_g_s values. The higher rigidity of C_5_N may lead to greater steric compression in the insertion, causing lower activities and increasing the probability of σ-bond metathesis and chain termination. Terpolymers containing DCPD have an unreacted double bond, for each DCPD inserted, available for post-polymerization reactions, which may lead to functionalized polymers.

In conclusion, we have shown that it is possible to obtain ethylene-norbornene and substituted cycloolefin terpolymers: activities, termonomer content, molar masses and thermal characteristics can be tuned by selecting the proper metallocene and termonomer structure.

## Figures and Tables

**Table 1:** Assignment of ^13^CNMR chemical shifts for carbons of poly(E-*ter*-N-*ter*-5-pentyl-2-norbornene), poly(E-*ter*-N-*ter*-5-octyl-2-norbornene) and poly(E-*ter*-N-*ter*-DCPD).

Region	ppm	Poly(E-*ter*-N-*ter*-C5N)	Poly(E-*ter*-N-*ter*-C8N)	Poly(E-*ter*-N-*ter*-DCPD)
		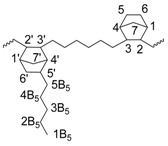	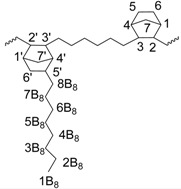	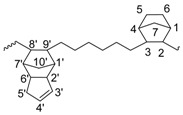
A	12.03 to 12.05	*1B_5_*	*1B_8_*	
	20.67 to 20.90	*2B_5_*	*2B_8_*	
B	25.00 to 36.25	*C5/C6*; *C6'*; *CH_2_ (E)*; *3B_5_*; *4B_5_*; *C7*; *C7'*; *5B_5_*	*C5/C6*; *C6'*; *CH_2_ (E)*; *3B_8_; 4B_8_*; *5B_8_*; *6B_8_*; *7B_8_*	*C5/C6; CH_2_ (E); C7;*
			*C7*; *C7'*; *8B_8_*	
	33.4			*C5'*
	34.35			*C10'*
C	36.26 to 50.00	*C1/C4*; *C1'/C4'*	*C1/C4*; *C1'/C4'*	*C1/C4*; *C1'/C7'*; *C2/C3*; *C8'/C9'*
		*C2/C3*; *C2'/C3'*; *C5'*	*C2/C3*; *C2'/C3'*; *C5'*	
	40.91			*C6'*
	51.72			*C2'*
	128.88			*C4'*
	130.64			*C3'*

**Table 2:** Poly(ethylene–*ter*–norbornene–*ter*–termonomer) using Catalysts **1** and **2**.

Entry ^a^	Catalyst	Termonomer	N/E/Termonomer ^b^	Activity (kg/mol h atm)	N (mol %) ^c^	Termonomer (mol %) ^c^	Cycloolefin content (%)	*T*g ^d^	*M*_w_ (kg/mol) ^e^	*M*_w_/*M*_n_^e^	DP ^f^
1	1	/	2/1/0	2,000	31.0	0	31.0	66	370	1.6	4,860
2	1	/	4/1/0	1,830	39.6	0	39.6	100	461	1.7	5,300
3	1	1-Octene	4/1/1	1,020	38.1	3.0	38.1	25	11	1.8	108
4	1	1-Octene	8/1/1	370	42.5	2.6	42.5	66	11	1.7	110
5	1	C_5_N	4/1/1	100	19.8	3.4	23.2	76	70	2.2	434
6	1	C_8_N	4/1/1	150	22.0	7.0	29.0	64	66	2.2	535
7	1	DCPD	4/1/1	120	30.4	3.6	34.0	101	83	1.6	1,022
8	2	/	2/1/0	1,920	36.6	0	36.6	87	157	2.1	1,433
9	2	/	4/1/0	1,700	45.0	0	45.0	129	195	2.1	1,649
10	2	/	8/1/0	1,230	53.7	0	53.7	164	220	2.4	1,439
11	2	1-Octene	4/1/1	1,400	45.8	7.3	45.8	36	19	1.9	151
12	2	1-Octene	8/1/1	500	48.2	6.0	48.2	86	35	1.7	309
13	2	C_5_N	4/1/1	380	41.5	6.7	48.2	119	29	1.7	268
14	2	C_8_N	4/1/1	880	37.4	8.7	46.1	83	164	1.6	1,536
15	2	DCPD	4/1/1	550	44.7	6.6	51.3	136	126	2.5	783
16	2	C_5_N	8/1/1	290	47.5	6.1	53.6	139	21	1.6	199
17	2	C_8_N	8/1/1	1,000	46.3	6.3	52.6	131	125	1.6	1,103
18	2	DCPD	8/1/1	180	53.8	3.5	57.3	152	91	2.1	640

^a^ Polymerization conditions: [catalyst] = 4 μmol; ethylene pressure = 1 atm; temperature = 50 °C; time = 15 min; volume = 0.1 L; [Al]/[Zr] = 2000; ^b^ Feed molar ratio; ^c^ Norbornene and termonomer mole percent determined by ^13^C NMR; ^d^ Determined by DSC; ^e^ Determined by SEC in *o*-dichlorobenzene by using standard polystyrene; ^f^ DP = *M*_n_/*M*_0_, where *M_0_* = molar mass of the monomer and *M_n_* = number average molar mass.

**Table 3:** ^1^H NMR data relative to the various chain unsaturations in the investigated terpolymer samples.

Structure	Notation	^1^H NMR (ppm)	Structure	Notation	^1^H NMR (ppm)
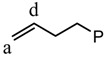	Vy-1	a: 4.80 to 4.93d: 5.72	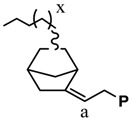	Nx-2	a: 5.38 to 5.39
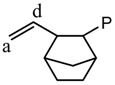	Vy-2	a: 4.74 to 4.85d: 5.69	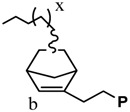	Nx-1	b: 5.56 to 5.57
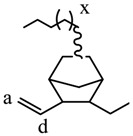	Vyx-2	a:4.74 to 4.85d: 5.63	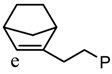	N-1	e: 5.44 to 5.50
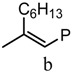	O-1	b: 5.11 [44]	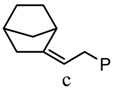	N-2	c: 5.30
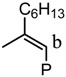	O-2	b: 5.09 [44]	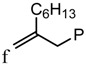	O-4	f: 4.68 [44]
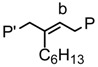	O-3	b: 5.08 [44]	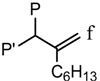	O-5	f: 4.73 [44]

## Supplementary Materials

The following are available online at www.mdpi.com/2073-4360/8/3/60/s1, Figure S1: ^13^C NMR spectrum (108.58 MHz, C_2_D_2_Cl_4_, 103 °C) of poly(E-*ter*-N-*ter*- C_5_N), sample prepared by **1** (Table 2, Entry 5), Figure S2: ^13^C NMR spectrum (108.58 MHz, C_2_D_2_Cl_4_, 103 °C) of poly(E-*ter*-N-*ter*-C_8_N), sample prepared by **1** (Table 2, Entry 6), Figure S3: ^13^C NMR spectrum (108.58 MHz, C_2_D_2_Cl_4_, 103 °C) of poly(E-*ter*-N-*ter*-DCPD), sample prepared by **1** (Table 2, Entry 7).
