# Supplementary material for: Terpolymerization of Substituted Cycloolefin with Ethylene and Norbornene by Transition Metal Catalyst
Source: Polymers (Basel). 2016 Feb 26;8(3):60. doi: 10.3390/polym8030060 (PMC6432560; doi:10.3390/polym8030060)
Supplement: Supplementary file 1 [file polymers-08-00060-s001.pdf]

Supplementary Materials: Terpolymerization of Substituted Cycloolefin with Ethylene and Norbornene by Transition Metal Catalyst

Laura Boggioni, Nella Galotto Galotto, Fabio Bertini and Incoronata Tritto

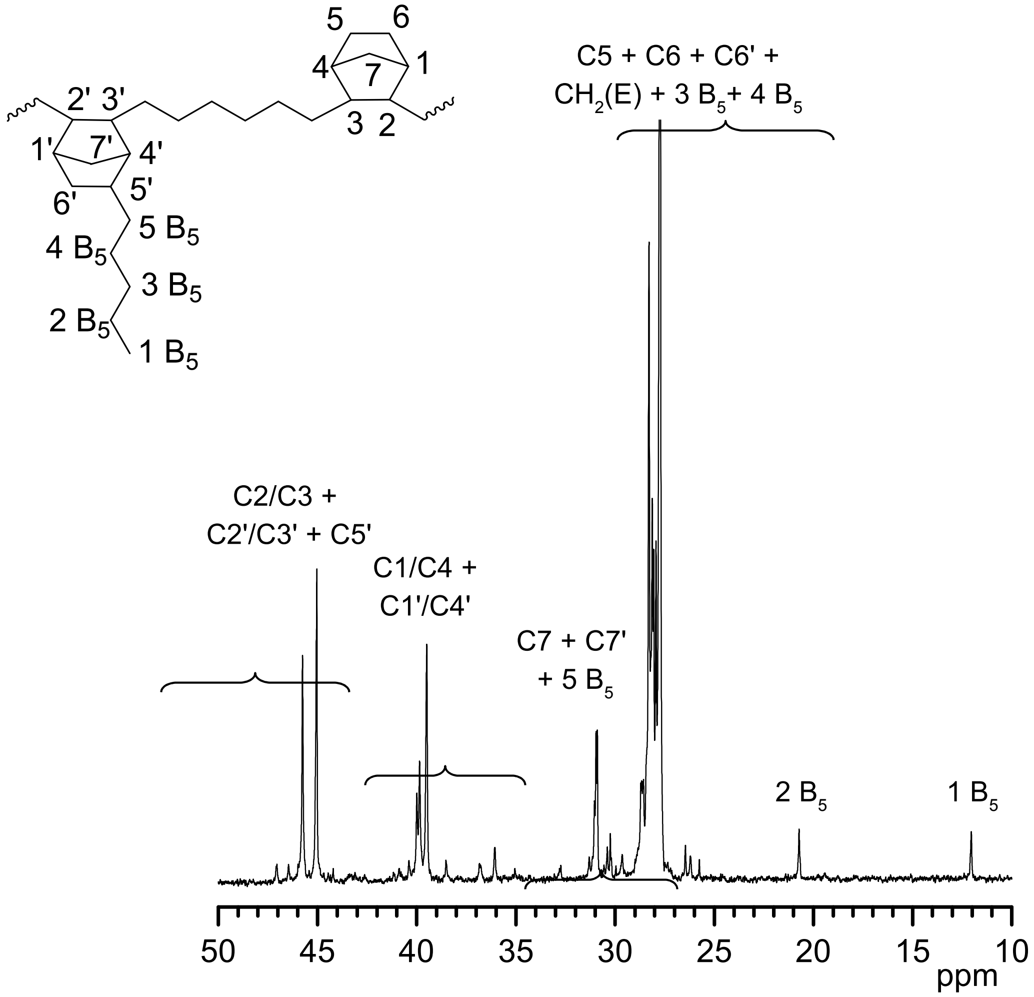

Figure S1. ^{13}C NMR spectrum (108.58 MHz, $C_2D_2Cl_4$, 103 °C) of poly(E-*ter*-N-*ter*-C5N), sample prepared by **1** (Table 2, entry 5).

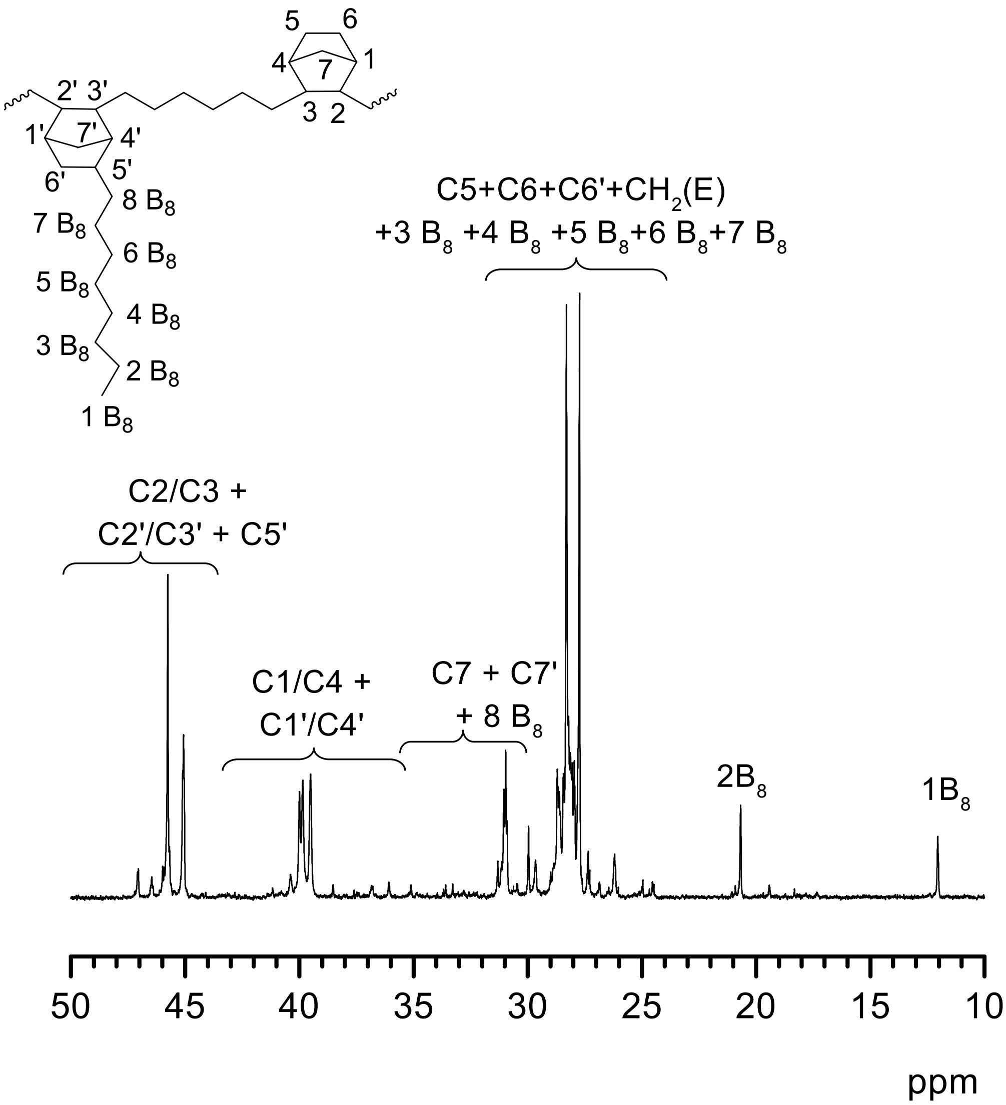

Figure S2. 13C NMR spectrum (108.58 MHz, $C_2D_2C_{14}$, 103 °C) of poly(E-*ter*-N-*ter*-C8N), sample prepared by 1 (Table 2, entry 6).

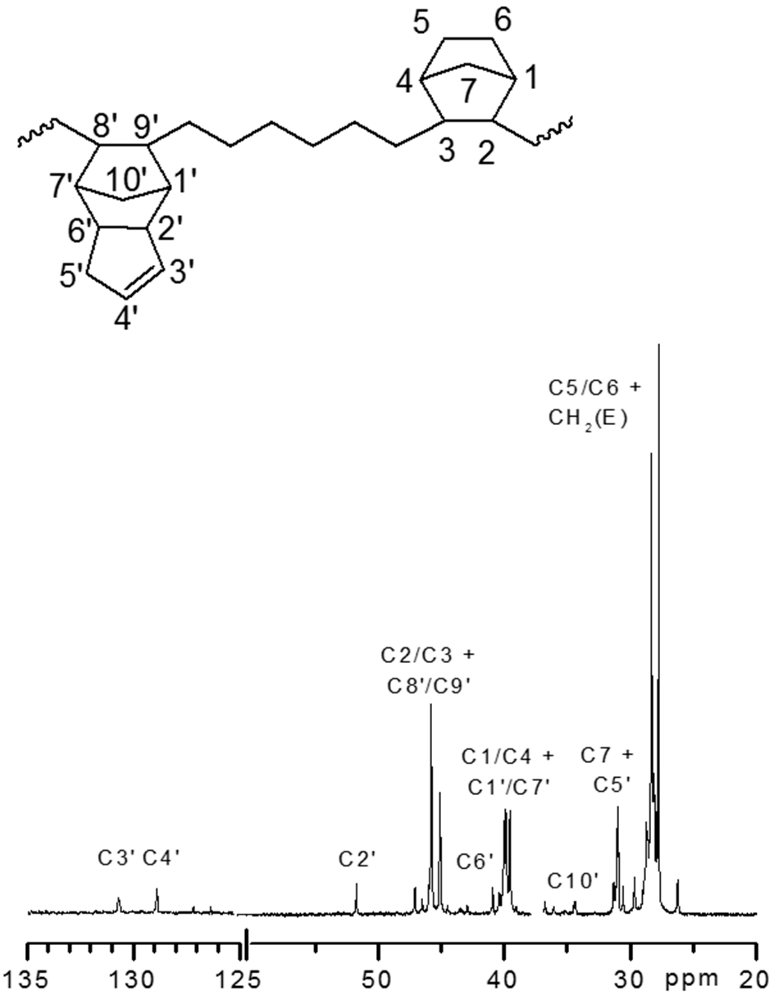

Figure S3. ^{13}C NMR spectrum (108.58 MHz, $C_2D_2Cl_4$, 103 °C) of poly(E-*ter*-N-*ter*-DCPD), sample prepared by 1 (Table 2, entry 7).

© 2016 by the authors; licensee MDPI, Basel, Switzerland. This article is an open access article distributed under the terms and conditions of the Creative Commons by Attribution (CC-BY) license (http://creativecommons.org/licenses/by/4.0/).
